# Risk Assessment of Transmission of Sporadic Creutzfeldt-Jakob Disease in Endodontic Practice in Absence of Adequate Prion Inactivation

**DOI:** 10.1371/journal.pone.0001330

**Published:** 2007-12-26

**Authors:** Nadège Bourvis, Pierre-Yves Boelle, Jean-Yves Cesbron, Alain-Jacques Valleron

**Affiliations:** 1 Université Pierre et Marie Curie-Paris6, Unité de Recherche Epidémiologie-Systèmes d'information-Modélisation, UMR S 707, Paris, France; 2 INSERM, U707, Paris, France; 3 Assistance Publique-Hôpitaux de Paris (AP-HP), Unité de Santé Publique, Hôpital St Antoine, Paris, France; 4 Laboratoire Adaptation et de Pathogénie des Micro-organismes, Université Joseph Fourier, UMR 5163, Grenoble, France; 5 Centre National de la Recherche Scientifique (CNRS), UMR 5163, Grenoble, France; 6 Centre hospitalier universitaire (CHU) de Grenoble, Laboratoire d'Immunologie, Grenoble, France; Yale University, United States of America

## Abstract

**Background:**

Experimental results evidenced the infectious potential of the dental pulp of animals infected with transmissible spongiform encephalopathies (TSE). This route of iatrogenic transmission of sporadic Creutzfeldt-Jakob disease (sCJD) may exist in humans via reused endodontic instruments if inadequate prion decontamination procedures are used.

**Methodology/Principal Findings:**

To assess this risk, 10 critical parameters in the transmission process were identified, starting with contamination of an endodontic file during treatment of an infectious sCJD patient and ending with possible infection of a subsequent susceptible patient. It was assumed that a dose-risk response existed, with no-risk below threshold values. Plausible ranges of those parameters were obtained through literature search and expert opinions, and a sensitivity analysis was conducted. Without effective prion-deactivation procedures, the risk of being infected during endodontic treatment ranged between 3.4 and 13 per million procedures. The probability that more than one case was infected secondary to endodontic treatment of an infected sCJD patient ranged from 47% to 77% depending on the assumed quantity of infective material necessary for disease transmission. If current official recommendations on endodontic instrument decontamination were strictly followed, the risk of secondary infection would become quasi-null.

**Conclusion:**

The risk of sCJD transmission through endodontic procedure compares with other health care risks of current concern such as death after liver biopsy or during general anaesthesia. These results show that single instrument use or adequate prion-decontamination procedures like those recently implemented in dental practice must be rigorously enforced.

## Introduction

Creutzfeldt–Jakob disease (CJD) was first described in the 1920s[1]. This rare neurodegenerative disease classically starts as a progressive dementia and leads to death within 6 months. The clinical diagnosis must be confirmed by histological analysis of the brain. There are four categories of CJD: 1) familial (fCJD) has a very low incidence of 1×10^−7^/year; 2) sporadic (sCJD) has an incidence in the range of 1–2×10^−6^/year; 3) new variant (nvCJD) caused by the agent of the bovine spongiform encephalopathy (BSE) and discovered in 1996[2]; and 4) iatrogenic (iCJD).

The first documented iCJD case, reported in 1977, was caused by the reuse of contaminated neurosurgery instruments[3]. Since then, 267 iCJD cases have been ascertained, following human growth hormone (hGH) injection, dura mater grafts, corneal transplants, neurosurgery, gonadotropin administration, and stereotactic EEG[4]. The last EuroCJD report [5] summarized CJD surveillance in 11 European countries over a mean duration of 14.4 years and reported 195 iCJD cases (out of a total of 6962 CJD cases), among which 143 were caused by hGH injection and the rest by dura mater grafts (n = 50) and corneal transplants (n = 2). The cases reported as iatrogenic in the surveillance systems were only those for which the route of transmission could be confirmed. Thus, it cannot be excluded that other iCJD cases could go unnoticed and be reported as sCJD. Several case–control studies investigated this possibility and a positive association between the total number of surgical interventions undergone and the risk of developing sCJD was found in several instances [6]–[8]. Although no specific procedures could be identified, those epidemiological findings strongly suggest that iatrogenic transmission of CJD may be, or may have been, much more widespread than currently seen in surveillance systems. This possibility is further supported by several pieces of evidence. First, tissue infectivity–or the ability of the sCJD pathogen in a tissue to cause infection–is not restricted to the central nervous system. Recently, the pathological form of the prion protein (PrP^sc^) was found in the spleen and skeletal muscles of sCJD patients [9] and their olfactory epithelium [10]. In sCJD-infected primates, a broad range of tissues, including peripheral nerves, was shown to harbour PrP^sc^ at levels higher than previously considered [11]. Thus, the number of procedures that can be considered at risk of TSE transmission is much higher than previously thought. The individual risk associated with these procedures may be low, but if these are performed on millions of patients the iatrogenic transmission may become of concern. Second, the existence of an infective state before symptoms appear is suggested by animal experiments [12]–[15] and clinical reports. Today, because no reliable diagnostic tool is available, detecting infectious carriers is impossible. Therefore, the numbers of potentially infectious subjects who may be infectious could be much higher than the figures of CJD incidence indicate. Third, decontamination procedures routinely used in the past were ineffective against the CJD agent [16]. Although autoclaving is effective for prion decontamination [17], the level of compliance with such practice in healthcare settings is unknown.

For all these reasons, it is not implausible that individuals were contaminated in the past, and could continue to be so if the proper decontamination procedures are not carried out and instruments reused. The low incidence and very long incubation period of CJD impairs the chance of a direct observation of these risks. Should observation be possible, it might occur at a time when it would be too late to efficiently intervene. Even if one chose to wait another dozen years to carry out a case control study, there is no guarantee that the risk could be identified if exposure is excessively common and experienced by virtually everybody over time. Furthermore, negative case control studies may not rule out the existence of a risk. A recent paper studying the risk of sCJD transmission through ophthalmic surgery makes this point clearly: a well designed case-control study did not evidence a significant increase of the risk of CJD after surgery, yet the conclusion insists on reinforcing proper decontamination procedures[18].

Finally, iCJD risk assessment poses a practical challenge for public health. Experts may be asked about the existence and potential magnitude of the risk. However, this derivation follows a “black box” approach. Mathematical modelling is the only scientific method to answer the question because it forces to state the different components contributing to the risk, to gather all available knowledge on these components, and to explicitly describe how the risk is computed.

Herein, we report our assessment of iatrogenic transmission of CJD during endodontic treatment (ET). Several characteristics explain the choice of this procedure for iCJD risk assessment. First, ET involves contacts with the richly innervated dental pulp, a tissue whose infectivity was demonstrated in animal models (intraperitoneal injection in hamsters) [19]. The intradental route of inoculation was also shown to efficiently transmit the disease [19]. It must however be stressed that no evidence supports the presence of PrP^sc^ in the dental pulp of sCJD patients. It was reportedly absent in patients who died of sCJD, but the minute amount of material available and insensitivity of the assays considerably limited the conclusiveness of those findings [20], while raising a lot of suspicion. Second, ET instruments are reused by the vast majority of practitioners [21]. They are known to be particularly difficult to clean and decontaminate because of their small size and complex surface structure, and, indeed, residues of proteic material are visible on most instruments after usual cleaning procedures [21]. Third, the frequency of exposure to ET is very high, in the order of 1 procedure/10 persons/year [22], [23]. This is a typical example of a procedure that virtually everybody in a population has experienced, which impairs any hope to get a risk assessment with a case-control study, would it be in decades.

In this study we provide an estimate of the iatrogenic risk of sCJD transmission in current and past endodontic practice, assuming that prion infectivity of dental pulp is real. We estimated whether–in the absence of effective decontamination procedures, as was the case during most of the 20^th^ century–these hypothetical transmissions could have put patients at risk, and whether that risk is sufficiently high to have initiated a small an epidemic process, defined as a process in which the secondary number of infected cases is larger than 1.

## Materials and Methods

### Description of ET and model implication

In ET, also called root canal treatment, the infected dental pulp is removed and then replaced by a filler. Before beginning the procedure, a rubber sheet, called a rubber dam, is stretched around the tooth and is held in place by a small clamp. Then, the pulp is accessed by drilling through the tooth into the pulp chamber. Most of the cleaning process is then accomplished with root-canal files or reamers. These have the structure of a screw (25 mm long, 0.08–0.8 mm diameter), with a spiral cutting edge on the surface. A series of these instruments, each with a slightly larger diameter, are used during the treatment, being bumped up and down, and with a simultaneous twisting motion, so as to scrape out and reshape the root canal. This action is traditionally performed manually; however, special dental drills can also be used. Once the tooth has been thoroughly cleansed, the canal is filled, most commonly with a rubber compound (gutta-percha) usually in conjunction with a sealer paste. These are inserted into the newly cleansed and reshaped root canal with a dental drill. Finally, the hole in the tooth created at the beginning of the procedure is also filled.

### Risk of sCJD transmission associated with instrument reuse

**Figure 1:**
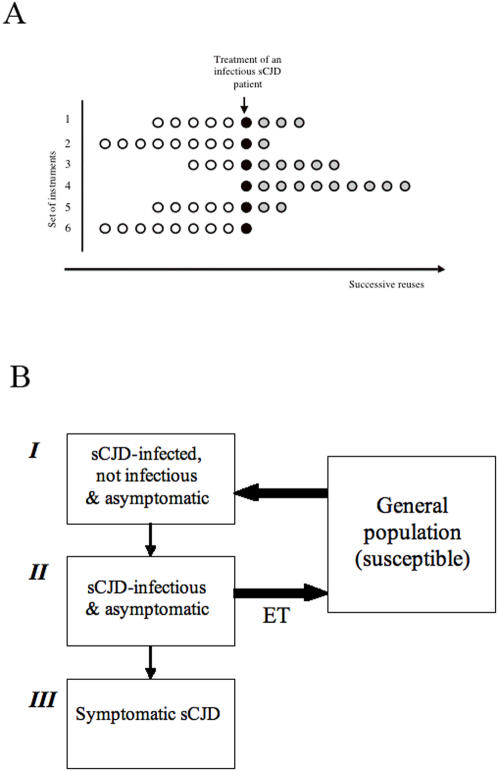
Pattern of use of ET instruments and natural history of sCJD transmission. a–In this example of ET, 6 files were used in an infectious sCJD patient. The history of each file is represented horizontally from left to right. At the time of ET (•), the total number of future reuses varies from one instrument to the other (here between 8 and 10). For example, instrument 4 was used for the first time on the infectious sCJD patient, and will be reused 9 times in subsequent patients. In contrast, instrument 6 is never reused after being contaminated. b–Flow chart of sCJD transmission and natural history in our model. The sCJD incubation period is divided into three phases: I, the infected individual is neither infectious nor symptomatic, II, the patient becomes infectious, but remains asymptomatic: this is the only period when iatrogenic transmission is possible. III, the patient is symptomatic and CJD has been diagnosed so that the patient MUST be treated with single-use instruments and will not infect others.

We postulated here that CJD transmission could be mediated by the reuse of endodontic instruments, because they are typically reused. If such an instrument were to be used on a new patient after having been used in an asymptomatic infectious patient, and if the cleaning/autoclaving of the instrument had not been 100% effective, a risk of transmission would exist. Figure 1 describes the typical pattern of ET instruments use in the treatment of an infectious sCJD patient, and the natural history of CJD transmission.

### Endpoints of the risk assessment

We quantified the risk of CJD transmission during ET from an individual and population perspectives. For the former, we estimated the risk for a patient of becoming infected during a root-canal treatment. For the latter, we quantified the population risk by the mean number of secondary cases after treatment of an infectious CJD patient (*SC*), the so called reproduction rate of the transmission process (denoted *R*).

### The risk of transmission model

The risk assessment of transmission depends upon the ten parameters listed in Table 1. The computation of the risk was carried out in five steps.

**Table 1:** The 10 components considered in the estimation of risk of iatrogenic sCJD transmission.

Risk component		Ranges used in the model	References
1. Brain infectivity	*BI*	10^7^–10^9^ human intracerebral *ID*_50_/g of brain tissue	From expert consensus based on Brown's unpublished data (2000) [25], [37]
2. Brain infectivity to pulp infectivity ratio	*BPR*	10^−3^–10^−4^	From experimental data in non-human primates infected with sCJD [11].
3. Mass remaining on endodontic instruments after cleaning	*M*	2.5–316 *μ*g	Experimental data on procedures for decontamination of dental instruments in the UK [21]
4. Agent infectivity reduction by autoclaving	*AIR*	10^−3^–10^−5^	[25]
5. Fraction of remaining material that detaches from instrument and inoculates the next patient	*F*_M_	1–10% of remaining mass M	As a working hypothesis in [25]
6. Total number of times of a single endodontic instrument is used	*U*	8–10 times	From expert consultation [25]
7. Number of distinct instruments used throughout the endodontic treatment of a tooth	*N*	6	From expert consultation [25] and oral personal communication from Pr Kleinfinger, Association Dentaire Française
8. Duration of infectious period in sCJD patients	*DI*	0.8–16 years	*DI* was taken as last 40% of the incubation period [38], which varies from 2 to 40 years [30]
9. Incidence of sCJD in the general population	*I*_GP_	0.8–2.2 cases/million/year	EuroCJD data for 2004 [5]
10. Frequency of endodontic treatment in the general population	*ET*_GP_	0.11–0.13 procedures/year/person	Personal communication from Pr Löst, European Society of Endodontics, epidemiological data [22], [23]

The load *L*, i.e., the 50% infectivity dose (*ID*_50_) remaining on an instrument after use in an infectious sCJD patient, cleaning and autoclaving (parameters 1–4, table 1) is given by the equation:
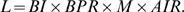
The infectivity load, *IL*, that is the real dose inoculated into a patient treated with a contaminated instrument bearing a load *L* is given by: *IL* = *L*×*F_M_* (parameter 5, table 1). After inoculation, the probability of infection for this patient is given by a dose response function ϕ. A meta-analysis of experimental data on scrapie indicated that the dose–response curve for TSE was S-shaped [24]. We therefore selected two dose–response functions for use in the model: *ϕ_1_* is 0 below 10^−1^ *ID*_50_, linear on the log_10_ scale between 10^−1^ and 10^1^ *ID*_50_, and 1 above; while *ϕ_2_* is 0 below 10^−2^ ID50, then linear on the log_10_ scale between 10^−2^ and 10^2^ *ID*_50_ and 1 above.

The estimation of the number of secondary cases (*SC*) depends on the number *N* of instruments used per procedure and on the infectivity load remaining on those instruments after each reuse. We assumed that the maximum number of times any of the *N* instruments is used in the treatment of an infectious sCJD patient was *U*. The number of reuses of a contaminated instrument may therefore range from 0 if the instrument was not used after the sCJD patient, to *U*–1 if it was first used on the sCJD patient. We assumed that, for a patient undergoing ET, an instrument would have been previously used *u* times with *u* uniform on the set {0, 1,…, *U*–1}. Then, according to steps 1 and 2, the average number of secondary cases *SC* contaminated by the initial procedure in the CJD patient is:
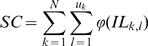
where *u_k_* is the number of reuses of the *k* ^th^ instrument, and *IL_k,l_* the real dose inoculated from the *k* ^th ^instrument on its *l* ^th^ reuse. The latter was calculated as:
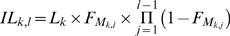
where *L_k_* is the initial load of the *k^th^* instrument, and 1-*F_M k,j_* is the fraction of infectivity load lost by the *k* ^th^ instrument during its *l* ^th^ reuse.

The risk of sCJD transmission was estimated considering the two exclusive possibilities that a given instrument does not contaminate during ET: either the instrument was never exposed to an infectious patient, or the instrument was exposed to an infectious sCJD patient, but infection failed to occur during this particular contact because the inoculum was too small. Taking both possibilities into account, the probability *p* of not being infected by an instrument during an ET was given by:
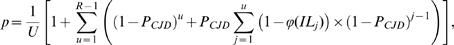


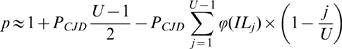
where *P_CJD_* is the (small) probability that a patient is incubating sCJD, *u* is the number of times the instrument was used prior to the patient, and *I_j_* is the infectivity load transferred from an instrument contaminated *j* uses earlier. The probability to be infected by one of the *N* instruments used in one procedure is 1−*p^N^*. Taking into account that P_CJD_ is small, we derived 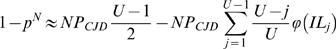.

This formula takes into account that a typical instrument has been used 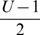 times before the patient, therefore the probability that at least one instrument was infected is approximately 
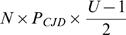
 where *P_CJD_* is the prevalence of infectious CJD cases in the population.

The cumulated number of secondary cases over the infectious lifetime of a sCJD patient is the reproduction number of the ET transmission, and is given by *R* = *SC*×*ET_GP_*×*DI*, where *SC* is the number of secondary cases following an ET episode calculated above, *ET*_GP_ is the frequency of ET and *DI* is the duration of the infectious period. The incidence, *I*_GP_, i.e. the number of newly infected person per year, is given by:
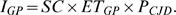



### Uncertainty analysis

All the parameters used in the risk assessment were given plausible ranges (Table 1).

We carried out Monte Carlo simulations, with 1,000,000 computations of the model endpoints, each time with a set of parameters sampled in a rectangular distribution within the proposed ranges.

## Results

**Figure 2:**
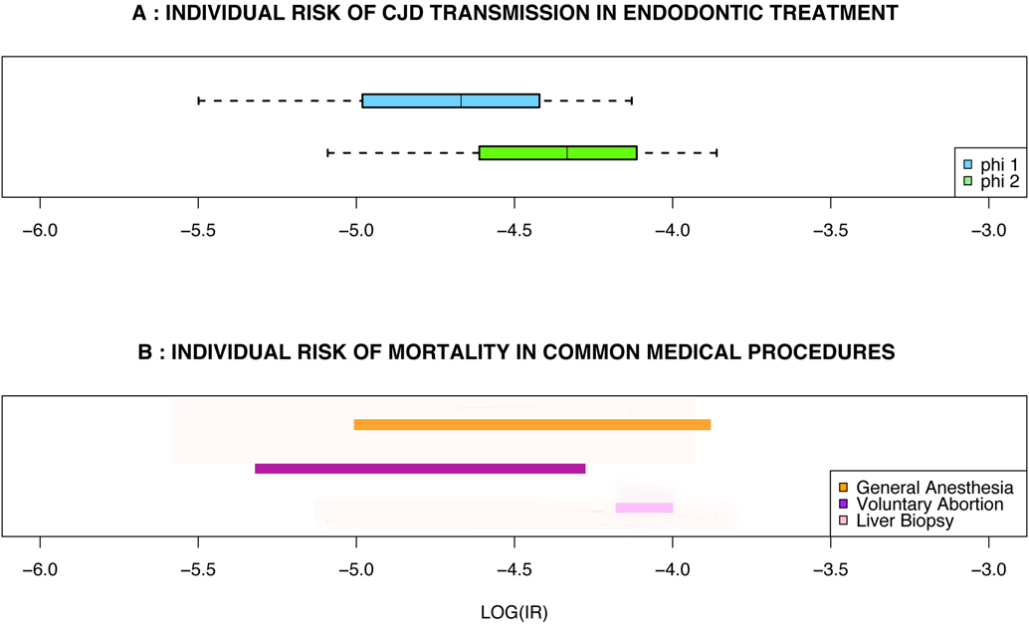
Individual risk assessment of sCJD transmission during ET, using dose–effects functions ϕ_1_ ( blue) and ϕ_2_ ( green). A: median (vertical line within the box), the 95% CI (T bars), and the 25^th^–75^th^ percentiles (left and right borders of the box). B: risks of iatrogenic mortality associated with other procedures.

There was a non zero risk of being infected with sCJD during ET under all hypotheses of dose–effect relationship. With *ϕ*_1_, the estimate of the mean individual risk was 2.8/100,000 (95% CI: 0.34/100,000–7.5/100,000), with *ϕ*_2_ it was 5.6/100,000 (95% CI: 0.86/100,000–14/100,000). The ranges of uncertainty were large (Figure 2) reflecting those of the model parameters. 

**Figure 3:**
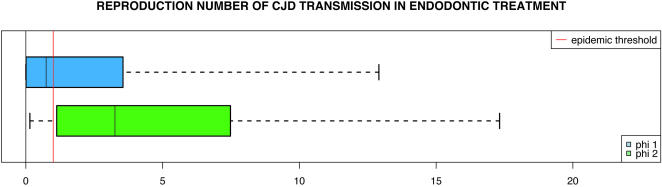
Population risk assessment of sCJD transmission during ET, using dose–effects functions ϕ_1_ ( blue) and ϕ_2_ ( green). See the legend to figure 2 for the description of the box plots. The vertical line passing through both plots is the epidemic threshold (R>1).

The basic reproduction rate *R* was also estimated (Figure 3). We decided to express the likelihood of the epidemic potential of ET with the dose–effect functions *ϕ*_1_ and *ϕ*_2, _using the percentages of cases when *R*>1. These proportions were found respectively equal to 47% and 77%. Ranges of possible values for R were broad (respective 95% CI of 0–12.9 and 0.14–17.3).

The model was also used to estimate the median number of secondary infections that could result from a single endodontic treatment that would be performed in an infectious sCJD patient. With dose-response functions *ϕ*_1 _this is 0.86 (95% CI of 0–10.8) and 4.1 (95% CI of 0.24–13.9) with *ϕ*_2_.

Finally, all computations were repeated, assuming that autoclaving was extensively used in dental practice and effective at decontamination. The estimates were virtually equal to zero in all instances. With *ϕ*_1_ and *ϕ*_2_, the means of individual risks were 4.6×10^−16^ and 2.0×10^−10^ and the respective annual incidences of secondary cases were 4.6×10^−17^ and 1.2 10^−11^.

## Discussion

The results of this modelling approach show that the risk of sCJD transmission due to the reuse of instruments during ET may not be ignored in absence of effective prion-decontamination procedures.

How should our conclusions be used in a public health assessment? First note that this risk is already of concern to national health agencies as well as to health professionals [25]–[27]. Our work makes it possible to go beyond a qualitative assessment, towards more quantitative predictions where all hypotheses are clearly stated. The conclusions of this approach may easily be updated as new data accrue.

The details of ET were obtained from the latest official reports in France and UK or from experts. We conducted a literature search to collect the best estimates available of the possible quantities of infectious material left on the instruments, and subsequently partially removed by the classical disinfection procedures used until the last years of the 20^th^ century. We obtained similarly estimates of the values of brain infectivity and of the ratio of brain infectivity to pulp infectivity. These parameters were obtained from animal experiments as they are clearly unknown in humans.

A comparable approach can be found in the HPA report on vCJD transmission in dentistry with two notable differences [25]. First, the route of instrument contamination and subsequent transmission considered in the HPA report was a very rare accidental process: the abrasion of tonsillar tissue during dental care. On the contrary, we considered the process of accessing the dental pulp during ET as certain, which obviously leads to a higher risk. Second, the HPA report considered infectivity of tonsils to be 10^6^–10^7^ i/c ID_50_ per gram, while we used dental pulp with a slightly lower range of infectivity from 10^4^-10^6^i/c ID_50_ per gram ( = BI* BPR).

The hypotheses we used concerning the relationship between the estimated inoculums and the probability of infection are obviously critical. In our assessment, we postulated that too small an inoculum (below 10^−1^ and 10^−2^ *ID*_50_ were considered with functions *ϕ*_1_ and *ϕ*_2_) would not lead to infection, and that there was a linear dose–response relationship above this threshold. This effectively complies with the “zero risk below a threshold” hypothesis rather than with the “single infectious particle” hypothesis. There is indeed experimental evidence that even very small quantities of infectious material may trigger infection in mice [13], [28], and this hypothesis was previously used in assessing decontamination procedures[29]. However, we adopted a more conservative risk estimate.

The duration of the infectious period of CJD is unknown but could be very long. We used as a reference the incubation period estimated from hGH iatrogenic cases [30]. To make comparison easier, and for want of better or more recent evidence, the duration of the infectious period relative to incubation was the same as in the HPA report, i.e. 40% of the incubation period [25].

Our risk assessment should have used the prevalence of infectious sCJD in those undergoing ET instead of that in the general population. Presumably, the former is the largest and the risk was therefore minimized. Indeed, children, who do not develop sCJD are taken into account in the general population estimate, when that in ET patients concerns only adults.

The ranges of values of risk assessment generated with our model were broad. They mirror the current lack of knowledge and the uncertainties concerning data and hypotheses. However, our model makes it clear that the ET of the 20^th^ century were not risk-free in terms of CJD. Therefore, our model suggests that patients may well have been contaminated at the end of 20^th^ century, and still be in the latency period and at risk of transmitting the disease.

The estimated individual risk of sCJD transmission during ET was low in our assessment. However, these values compared with the mortality rates in general anaesthesia [31], transcutaneous liver biopsy [32] or voluntary abortion [33] which are of concern in the modern health care.

We also studied the possible impact of ET at a population level and showed that there was a high probability that the reproduction rate *R* exceeded 1 in the absence of effective prion decontamination of the instruments: one of the conditions for the initiation of an epidemic process is fulfilled. To date, epidemiological surveillance data did not evidence such an epidemic process. However, would our hypothesis be true, the increase in incidence could remain modest and hard to identify for dozens of years because the incidence of sCJD is low, the incubation period long and in competition with all mortality causes present. CJD surveillance systems is too recent to show such trends.

Vacuum autoclaving and porous-load autoclaving for 18 min at 134°C are currently recognised as appropriate methods for prion decontamination, leading to a reduction of the infectivity load by of 3–5 log_10_ or more. According to our model, this decontamination would prevent CJD transmission in dental practice, even considering that the residual infectivity is not strictly reduced to zero. These methods are recommended in official reports in various countries. However, in a US study conducted in 1996 [34], only 53% of dentists used autoclaves to decontaminate root-canal files. In a survey conducted in France in 2004, only 79% of dentists used an autoclave [35]. The problem of correct use of the autoclaves and regular checking of their efficacy has also been raised by many authors in several countries [34]. A recent survey on dental practice also showed that other elementary precautionary measures against CJD transmission were not widely respected. For example, the vast majority of dentists did not actively seek out patients at-risk for any form of CJD (sporadic, iatrogenic or familial) [36]. Therefore, in the current situation and despite recommended decontamination procedures, the risk of sCJD transmission during dental care might still not be zero. In any case, our findings constitute a strong argument for the strict respect of the official recommendations on decontamination procedures in dentistry, and even suggest that the cost-benefit of single-use endodontic instruments should be re-evaluated.

The risk analysis approach we have used relies on a “problem dissection” in which all components to a risk are identified and linked to the available scientific data, knowledge, and expert opinion. It may be of help in other emerging diseases, when data on the natural history of the disease and transmission are still scarce and clinical events cannot be observed directly. In all these cases, the output of the work will always be questionable, because of the lack of data, but the strength of the method is that its results and final statements are refutable as data accrues.
